# Dissecting the Syndrome of Schizophrenia: Progress toward Clinically Useful Biomarkers

**DOI:** 10.1155/2011/614730

**Published:** 2011-06-18

**Authors:** Brian Dean

**Affiliations:** ^1^The Rebecca L. Cooper Research Laboratories, The Mental Health Research Institute, Locked bag 11, Parkville, VIC 3052, Australia; ^2^The Department of Psychiatry, The University of Melbourne, Parkville, VIC 3052, Australia

## Abstract

The search for clinically useful biomarkers has been one of the holy grails of schizophrenia research. This paper will outline the evolving notion of biomarkers and then outline outcomes from a variety of biomarkers discovery strategies. In particular, the impact of high-throughput screening technologies on biomarker discovery will be highlighted and how new or improved technologies may allow the discovery of either diagnostic biomarkers for schizophrenia or biomarkers that will be useful in determining appropriate treatments for people with the disorder. History tells those involved in biomarker research that the discovery and validation of useful biomarkers is a long process and current progress must always be viewed in that light. However, the approval of the first biomarker screen with some value in predicting responsiveness to antipsychotic drugs suggests that biomarkers can be identified and that these biomarkers that will be useful in diagnosing and treating people with schizophrenia.

## 1. Introduction

There is a growing recognition that the development of drugs with an increasing efficacy for reducing the psychotic symptoms of schizophrenia (Sz) is slowing and there has been little progress in developing treatments for the negative symptoms and cognitive deficits associated with the disorder [1]. In addition, it is now acknowledged that the current symptom-based diagnoses of Sz defines a syndrome of disorders which are likely to have diverse pathophysiologies [2]. This hypothesis is supported by the existence of treatment resistant Sz [3] which does not appear to be primarily associated with the dopaminergic systems of the human CNS because they are the primary targets of antipsychotic drugs [4]. The limitation of having to study the syndrome of Sz is now recognised as hampering progress toward a better understanding of the pathophysiologies of its component disorders [2] whilst having to provide treatments at the level of the syndrome is a major factor preventing the development of personalised medicine [5]. As with other forms of medicine, the solution to both of these problems would be to divide subjects with Sz into more biologically homogenous subgroups using well validated biomarkers as diagnostic tests [6] which in some cases may result in separating groups into endophenotypes [7]. To fulfil the requirement of an endophenotype, subjects clustered by the use of a specific biomarker must have a disorder which is heritable, be primarily state independent and, within families, the endophenotype and illness cosegregate [8]. The differences between dissecting a syndrome using a diagnostic test or into endophenotypes is discussed extensively elsewhere [9]. Briefly, both a biomarker that can be used as a diagnostic test and the characteristics or tests used to define an endophenotype must be detectably different in the large majority, if not all, the subjects within the category defined by the biomarker or an endophenotype.

This paper will focus on the evolving notion of biomarkers and on the progress toward developing biomarkers that will allow subjects with Sz to be divided into more homogeneous subpopulations as a foundation to better understanding their pathophysiologies and responsiveness to treatments.

## 2. The Evolving Concept of a Biomarker

**Figure 1:**
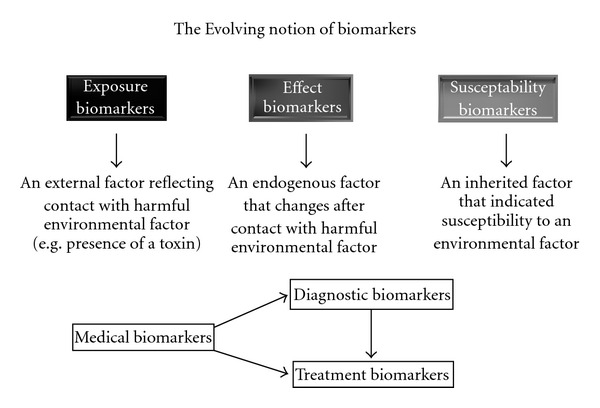
A schematic showing the evolving notion of biomarker utility from the inception of the concept of biomarkers to their use in medical research. This schema acknowledges that the discovery of diagnostic biomarkers is likely to also allow the separation of the different-component disorders in the syndrome of schizophrenia and that more tailored treatment of these component disorder will be possible. Hence diagnostic biomarkers, to a greater or lesser extent, are also likely to be treatment biomarkers.

The initial definition of a biomarker was not focussed on purely medical requirements but defined a measurable event occurring in a biological system that could inform on the general state of an organism, its life expectancy, or the well being of its environment (Figure 1) [10]. These concepts saw biomarkers divided into three categories: exposure biomarkers, effect biomarkers, and susceptibility biomarkers [11]. An exposure biomarker might be a xenobiotic or metabolites that reflect contact with a harmful environmental factor (e.g., toxin), an effective biomarker was an endogenous component of an organism that would change after exposure to an environmental factor whilst a susceptibility marker would be an inherited factor that provided a measure of susceptibility or sensitivity to an environmental factor. It was proposed that the major advantages of identifying biomarkers for these three categories were to elucidate pathogenic mechanisms, to improve etiologic classification of environmentally related disease and to allow an early recognition of contact with disadvantageous environmental factors [12].

More recently, in the medical arena, biomarkers are viewed as measurable factors that can enable diagnoses [13], inform on disease pathophysiology [13], or enable decisions to be made on the optimal treatment of an individual (personalised medicine) [14]. The value of well-validated biomarkers now has strong face validity as drug regulatory bodies have, for example, acknowledged that certain biomarkers do have predictive validity for treatment responsiveness in some forms of cancers [14]. It is also well recognised that well-validated biomarkers can differentiate component disorders within diagnostic syndromes and that the separation of the component disorders within a syndrome leads to rapid advances in understanding the underlying pathophysiologies of those disorders [13, 15, 16]. Existing experience in biomarker validation has shown this process to be a far from trivial undertaking. For example, perhaps the earliest biomarker was the sweet taste of urine first used by Thomas Willis to identify subjects with diabetes in 1674 [17]. However, it was not until 1949 that Himsworth proposed two disorders within the syndrome that could be delineated based on the presence or absence of insulin resistance [18]. Over time, the form of diabetes that was not associated with insulin resistance became known as insulin-dependent diabetes mellitus and is now recognised as an autoimmune disease [19] that can be treated with immunotherapy [20]. By contrast, the form of diabetes associated with noninsulin-dependent diabetes mellitus is now primarily defined by the presence of insulin resistance [21] and initial treatments usually involve lifestyle changes and drugs that can lessen tissue insulin resistance [22]. Research is still ongoing to better understand and develop better treatments for both forms of diabetes.

Given lessons learned in other discipline, those in the psychiatry arena must acknowledge that biomarker discovery and validation will be difficult and time consuming. However, the rewards following the discovery of reliable biomarkers will be significant advances in understanding the causes and treatment of the component disorders within diagnostic syndrome such as Sz.

## 3. Biomarker in Psychiatry

The identification of biomarkers has been one of the holy grails for psychiatric research for many years [23]. Given that biomarkers are not widely used for either diagnoses or treatment monitoring of psychiatric disorders, the challenge of identifying clinically useful biomarkers must remain a high research priority. Recently, biomarker discovery has seen a significant boost with the availability of  “omic” technologies that permit the large-scale rapid screening of DNA sequence (genome) [24], levels of gene expression (transcriptome) [25], levels of gene translation (proteome) [26], and levels of metabolic activity (metabolome) [27]. These technologies are now producing large data sets that could well be the foundation for identifying biomarkers that will be useful in diagnosis and directing treatment for serous psychiatric disorders such as Sz. Hence it would seem appropriate to review the progress that has been made towards identifying biomarkers that can be used to assist in the diagnoses and treatment of Sz.

## 4. Peripheral Biomarkers for Sz: Biochemical, Genetic, and Pharmacogenomic Approaches

The initial search for biomarkers focussed on peripheral tissue. This was partly a pragmatic approach but also reflected the reality that a peripheral biomarker would have immediate clinical utility. One suggested diagnostic biomarker for Sz was the presence of 3,4-dimethoxyphenylethylamine in the urine of subjects with the disorder [28]. Importantly, when first reporting evidence to support this hypothesis, the authors stated that experiments would be required to validate the use of urinary 3,4-dimethoxyphenylethylamine as a biomarker for Sz. These subsequent experiments proved that urinary 3,4-dimethoxyphenylethylamine was not a biomarker for Sz and that there was doubt as to whether the “pink spot”, by which the presence of 3,4-dimethoxyphenylethylamine was measured, was a measure of that chemical entity [29]. Thus, the outcome from the initial experiments that suggested a diagnostic biomarker for Sz had been identified was disappointing but the ensuing debate led to the realisation that “perhaps the time has now finally come to stop investigating “schizophrenics” en masse, and to concentrate on individuals” [29]. Importantly, it is still acknowledged that the study of the syndrome of Sz is a major impediment to identifying the causes of the symptoms associated with the disorder [2]. It is also now clear that it is unlikely that a single biomarker will be identified for any particular symptom such as psychoses [30]. These two hypotheses acknowledge that Sz is a complex syndrome, however it was hoped that an extensive study of the human genome in subjects with Sz would reveal DNA sequence variation that were associated with an increased risk of developing the symptoms experience by people with the disorder [31] and hence lead to biomarkers with which to diagnose the disorder. 

The notion that changes in DNA sequence could be associated with an increased susceptibility to Sz came from the recognition that the disorder was highly heritable [32]. The hypotheses that changes in the genome could be used to diagnose Sz gained significant support from the identification of susceptibility locus for Sz in chromosome 5 [33]. More extensive studies showed that there was not a susceptibility marker for Sz on chromosome 5 [34] but other studies have generated a large body of data that may lead to the identification of genetic biomarkers that could be used for either the diagnosis of Sz [35] or as an indicator of the best treatment for an individual with Sz with a particular genetic background (pharmacogenomics) [36]. Unfortunately, the failure of large-scale genomewide associations studies (GWAS) to identify strong genetic markers that can predict altered risk for Sz [37] suggests that no one genetic marker is associated with a major susceptibility risk for the disorder at the level of the syndrome. This has led to two proposals, one is that genetic associations with an altered risk for Sz can be discovered if larger cohorts are used to compensate for the search for low-effect size genetic associations [24] or that Sz does not result from genetic changes at the level of single nucleotide polymorphisms [37]. The failure to identify genetic markers that show an increased susceptibility for Sz may also support the argument that genetic testing may be more effective in identifying the risk of symptom severity level. This idea is supported by the reports of genetic markers associated with the severity of psychotic symptoms [38], cognitive deficits [39] and decline in IQ [40] in subjects with Sz. However, these findings need replication in large cohorts before they can be judged to be of clinical value. 

Although many genetic studies have focussed on identifying diagnostic biomarker, other studies have used pharmacogenomics approaches to identify changes in DNA sequence that may provide indicators of treatment responsiveness based on genetic background [41]. Pharmacogenomic studies have shown that genetic polymorphisms in the cytochrome P450 gene can influence antipsychotic drug treatment responsiveness [41]. The cytochrome P450 gene is critical in antipsychotic drug metabolism and hence can be an indicator of antipsychotic drug clearance which is often inversely related to drug responsiveness [42]. It has also been suggested that polymorphism in the dopamine D2 receptor [43, 44], dopamine D3 receptors [43], the serotonin 2A receptors [44], and catechol-O-methyl transferase [44] genes may be predictors of responsiveness to antipsychotic drugs and that polymorphisms in dopamine receptor genes and the serotonin 2C receptor gene may predict susceptibility for side-effects such as tardive dyskinesia and weight gain, respectively [43]. These findings have inherent face value as the proteins encoded by these genes are targeted by many antipsychotic drugs [45]. However, large-scale studies will be required to establish the clinical usefulness of these pharmacogenomics markers.

## 5. Biomarker for Sz: Blood Based Studies

Complimentary to studies using genetic material, predominantly obtained from white blood cells, there has been a long history of research trying to identify other blood-based markers for Sz. A lot of this research has focussed on the study of the human platelet which has some biochemical processes that are similar to those present in neurons [46, 47]. Many of the more recent studies have suggested that the functioning of the platelet may provide some indication as to likely antipsychotic drug responsiveness. For example, it has been suggested that low platelet serotonin 2A receptor levels prior to treatment may predict responsiveness to olanzapine [48] and that changes in dopamine uptake by platelets may provide a more generic indication of responsiveness to antipsychotic drug treatment [49]. Changes in basic platelet function, such as a blunted serotonin responsive aggregation [50] and neurotransmitter uptake [51], have also been suggested to be potential biomarkers for Sz. Finally, it has been reported that platelet functions such as dopamine uptake [52] and monoamine oxidase activity [53] may correlated with the severity symptoms such as delusions and auditory hallucinations, respectively. Unfortunately, many platelet-based findings need to be replicated in well-controlled studies involving large cohorts before their value as potential biomarkers can be fully assessed [54].

## 6. Biomarker for Sz: High Throughput Screening of Blood and Brain Gene Expression

Biomarker discovery research has been accelerated by the development of high-throughput screening techniques, such as microarrays, which allow levels of the expression of multiple genes in a tissue to be measured effectively in short-time frame [55]. The initial study using such technology to probe the transcriptome in human postmortem CNS suggested that Sz was a disorder of dysregulated synaptic-related gene expression [56]. This finding was interesting given the hypothesis that Sz is a disorder that only occurs in humans [57] and that one of the major differences between humans and other species, at the level of gene expression, are changes in synaptic-related gene expression [58]. In addition, other studies examining changes in the transcriptome in the CNS of subjects with Sz suggest that changes in gene expression in Sz vary with duration of illness [59, 60]. These findings raise the intriguing possibility that some biomarkers could be a measure of disease progression and may be useful in predicting changes in drug responsiveness that are known to occur with the progression of the disorder [61].

High-throughput screening presents the opportunity to bring together different findings relating to the pathophysiology of Sz that may prove seminal to biomarker discovery. Thus, microarray gene-expression studies using postmortem CNS tissue added to genetic studies using peripheral DNA (association studies) suggest a role of regulator of G protein signaling 4 in the pathophysiology of Sz [62]. This is an important finding in its own right but also serves to illustrate how a holistic approach using multiple technologies can serve to identify potential biomarkers for the disorder. In addition, combining results from studies using CNS and peripheral tissue is beginning to suggest that changes in CNS gene expression may, at least in part, be detected using peripheral tissue such as white blood cells. This hypothesis is supported by studies showing similar changes in gene expression levels for some genes in blood and brain tissue (e.g., B-cell translocation gene 1, glycogen synthase kinase 3*α*, major histocompatibility complex, class II, DR *β*1, heterogeneous nuclear ribonucleoprotein A3, selenium binding protein 1, and splicing factor, arginine/serine-rich 1 [63]) from subjects with Sz. These findings are particularly important as they indicate it is possible to translate biomarkers identified using postmortem CNS into clinically useful tools because they can be measured in peripheral tissue and that, conversely, some changes in peripheral gene expression may provide insight into changes in brain gene expression in the CNS of subjects with Sz. Such conclusions must still be tempered by acknowledging other findings from microarray studies suggesting that changes in gene expression in peripheral blood cells are not present in the CNS from subjects with Sz [64, 65].

There are now intriguing data that could support the use of peripheral changes in gene expression as biomarkers for Sz. For example, changes in levels of selenium binding protein 1 expression have been found in peripheral tissue [63] and in the CNS from subjects with Sz [66]. However, the study of selenium-binding protein 1 expression in blood has shown that levels of gene expression correlate with levels of psychotic symptoms independent of whether the blood donor had Sz of bipolar disorder (BD) [63]. These latter data suggest that changes in gene expression may be associated with symptom severity and be associated with phenotypes rather than diagnoses [67]. If that is the case, gene expression biomarkers may be confirming the suggestion that the time has finally come to stop investigating schizophrenia in isolation as suggested over four decades ago [29]. Rather, it may be time for a more holistic approach testing potential biomarkers on a gene by gene basis carefully assessing their diagnostic potential but also whether they are pointing to commonalities in genetic susceptibility to multiple psychiatric disorders.

## 7. Biomarker for Sz: High Throughput Screening of the Blood and Brain Proteome

Whilst changes in levels of gene transcription at the level of mRNA may be a biomarker for Sz it is now recognised that not all changes in the transcriptome translate into changes in levels of the encoded protein [59] and hence the physiological significance of altered gene transcription is not readily apparent. Given one goal of biomarker discovery is to improve knowledge of disease pathophysiology, high-throughput methodologies that allow the study of the human blood and brain proteome have been used to identify potential biomarkers for Sz. This approach has been validated by a recent report that 25% of subjects with Sz can be separated into a separate population based on the loss of the majority (*∼*75%) of their cortical muscarinic M1 receptors [68] which shows that levels of proteins can be used to identify subgroups of subjects within the syndrome of Sz. Moreover, the subjects with Sz with a marked loss in muscarinic M1 receptors have been shown to have altered muscarinic M1 receptor signalling [69] and increased levels of a microRNA that targets muscarinic M1 receptor mRNA to reduce protein translation [70] that are not present in people with Sz that do not have changes in muscarinic M1 receptors. These data support the hypothesis that (i) it should be possible to discover biomarkers for the identification of subgroups of subjects within the syndrome of Sz and (ii) once these subgroups of subjects have been identified it should be possible to understand their specific pathophysiologies. The findings on cortical muscarinic M1 receptors also argues against the position that the use of high-throughput technologies are essential for biomarker discovery in Sz [71].

A number of studies of the human proteome have claimed to identify biomarkers for Sz. One study using surface-enhanced laser desorption/ionization time of flight mass spectrometry and postmortem brain tissue suggests that there are at least 21 potential biomarkers (Table 1) that can be used to separate Sz and/or BD from controls [72]. Again these data suggest that not all biomarkers will always show diagnostic specificity but some may be useful in assessing common factors that cross psychiatric diagnostic boundaries. A study using postmortem brain tissue and the same technology to measure protein levels found that no single protein could be used as a diagnostic-specific biomarker for Sz [73] but that a cluster analysis of the intensities of several proteins were required to separate subjects with Sz with an accuracy of 70%. Thus, whilst initial outcomes from the use of surface-enhanced laser desorption/ionization time of flight mass spectrometry are encouraging, there is now a need for follow-up data to support the proposed proteins, or groups of proteins, as biomarkers for Sz; of course these studies should also advance understanding of the pathophysiology of the disorder.

Using a liquid chromatography/mass spectrometry approach 10 proteins (CD5 molecule-like (CD5L), IGHM immunoglobulin heavy constant mu (IGHM), coagulation factor XIII, B polypeptide (F13B), transferring (TF), apolipoprotein D (APOD), apolipoprotein A-1 (APOA1), apolipoprotein A-IV (APOA4), apolipoprotein A-II (APOA2), apolipoprotein C-I (APOC1), and alpha-2-HS-glycoprotein (AHSG)) have been identified as altered in serum from drug naïve subjects with Sz compared to controls [74]. Clearly the identification of changes in the blood proteome in drug naive subjects is significant as the effects of antipsychotic drug treatment would not be a confound. Significantly, APOD [75, 76], APOA1 [77–79], and TF [80, 81] have previously been identified as being altered in blood from subjects with Sz. In addition, APOA4 has been reported as altered in the CSF from subjects with Sz [82]. These findings obtained in protein-specific studies add to the hypotheses that blood proteins can be used as biomarkers in Sz.

Another recent approach to identify biomarkers for psychiatric disorders is a large study comparing proteins levels in plasma from subjects with Sz, major depressive disorders (MDD), and control subjects [83]. In this study biomarker identification relied on a technology that examined levels of multiple chemokines, cytokines, hormones, growth factors, and antigens. Significantly, the study showed a greater separation of samples from subjects with Sz from controls than was achieved for MDD and controls using cluster analysis. The proteins that most strongly allowed the separation of Sz from controls and MDD were chemokine (C-C motif) ligand 4 (CCL4), chemokine (C-C motif) ligand 5 (CCL5), chemokine (C-C motif) ligand 22 (CCL22), chemokine (C-X-C motif) ligand 5 (CXCL5), TIMP metallopeptidase inhibitor 1 (TIMP1), epidermal growth factor (EGF), serpin peptidase inhibitor, clade A (alpha-1 antiproteinase, antitrypsin), member 7 (SERPINA7), and KIT ligand (KITLG). Conversely, changes in blood levels of insulin (INS), alpha-2-macroglobulin (A2M), matrix metallopeptidase 9 (gelatinase B, 92 kDa gelatinase, and 92 kDa type IV collagenase) (MMP3) and tumour necrosis factor receptor superfamily, member 1B (TNFRSF1B) allowed the separation of MDD from controls and subjects with Sz. Finally, brain-derived neurotrophic factor (BDNF), serpin peptidase inhibitor, clade E (nexin, plasminogen activator inhibitor type 1), member 1 (SERPINA1), and APODA1 allowed the separation of Sz and MDD from controls but did not show diagnostic specificity.

Reviewing results from blood-based biomarker studies critically, it is notable that there is no agreement on potential biomarkers across two studies. This could be due to many uncontrolled variables across the studies as blood proteins levels are known to vary with multiple factors including time of day [84], menstrual cycle [85], and food intake status [86]. Hence, at some point those involved in blood biomarker discovery for Sz should attempt to come to a consensus with regard to standardisation blood collecting with regards to fasting status, time of bleed, and other critical factors which can add variability to blood protein levels, as has been advocated for biomarker discovery for other diseases [87].

Another approach to identifying potential biomarkers at the level of the protein has been to study the CNS proteome in subjects with psychiatric disorders [88]. Whilst the study of the human CNS proteome in Sz has been ongoing for over a decade [89, 90] only 16 gray matter proteins (aldolase C, fructose-bisulphate, creatine kinase (brain), dynamin 1, dihydropyrimidinase-like 2, enolase 1 *α*, enolase 2 (*γ* neuronal), glial fibrillary acidic protein, glutamate-ammonia ligase, guanine nucleotide binding protein *β* polypeptide 1, internexin neuronal intermediate filament protein *α*, and *N*-ethylmaleimide-sensitive factor) and 8 white matter proteins (dihydropyrimidinase-like 2, inositol-monophosphatase 1, neurofilament, light polypeptide, Parkinson Disease 7, 14-3-3 zeta, l-lactate dehydrogenase Chain B, and stathmin 1) have been reported as altered in the same direction in more than one study of the human proteome [91]. Of equal concern is that some of these proteins, such as dynamin 1 [92], *N*-ethylmaleimide-sensitive factor [93], glial fibrillary acidic protein [94], and 14-3-3 zeta [95], have been reported to be unaltered in the CNS of subjects with Sz when measured using Western blotting. However, it has to be acknowledged that current studies on the human CNS proteome have been completed using relatively small number of cases and this is an issue given the need for the adequately powered cohort sizes needed when attempting to identify clinically valid biomarkers in any form of disease [96].

## 8. Towards Clinically Useful Biomarkers for Sz

There is a high expectation that the discovery of biomarkers will greatly accelerate our understanding of the pathophysiology of syndromes such as Sz [2]. This high expectation brings the danger that “apparent” failures to identify useful biomarkers may lead to the perception that biomarker discovery is an impossible goal. However, biomarker discovery in Sz is still in its infancy and the likelihood of identifying biomarkers is dependent on a number of factors, not the least is the ongoing development of the high-throughput technologies that allow the probing of the human genome, transcriptome, proteome, and metabolome.

In this respect, deep sequencing is a new technology that will allow a much more comprehensive analysis of the human genome in much larger cohorts of subjects with disorders such as Sz [97] and this technology may be able to identify genetic biomarkers even in a complex syndrome such as Sz [31]. At the level of transcriptomics, an increasing knowledge of the complexities of gene expression is producing technologies that can probe mRNA at ever increasing levels of complexity [98]. In addition, there are efforts to refine existing technologies [99] and utilise new technologies [100, 101] to increase the proportion of the human proteome that can be effectively measured in blood, CNS, or other tissues.

The capacity to significantly increase the reach of proteomics technologies in psychiatric disease is illustrated by the application of a differential-detergent solubility, multi-gel approach to analysis of the human CNS proteome in tissue from subjects with Sz, BD, and control subjects [99]. This approach has led to the visualisation of approximately 3,800 protein spots in the acidic protein fraction from cortex and caudate-putamen, this is close to triple the number of protein spots visualised using crude homogenates as reported in most studies from subjects with Sz [99].

**Figure 2:**
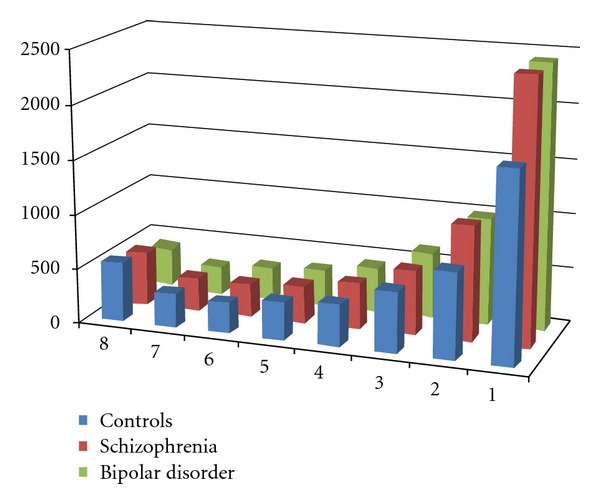
The number of protein spots visualised in Brodmann's area 46 from subjects with Sz, bipolar disorder, and schizophrenia after differential detergent solubilisation and separation across 5 two dimensional gels.

This methodology has now been applied to studying the acidic and basic proteins in Brodmann's area 46 (BA 46) from subjects with Sz, BD, and controls with 8 subjects in each cohort. Using this methodology, a total of 8152 protein spots were visualised across all of the cases studied, of which 5590 (69%) were present in two or more cases. Significantly, as is the case in some studies, if data analyses was limited to the study of protein spots visualised in every case then only 245 protein spots (3%) would be included for analyses. This would exclude the analyses of spots that seemed to have a significant variation with diagnoses (e.g., present in >75% of controls and <25% of cases with Sz). At the level of disease cohorts, the highest number of protein spots were visualised in tissue from subjects with Sz (*n* = 5917), followed by tissue from subjects with BD (*n* = 5746) and the fewest spots were detected in tissue from control subjects (*n* = 4946). These data are interesting as they suggest that subjects with Sz and BD expressed more CNS proteins or have overall higher levels of lower abundance CNS proteins, than do control subjects. Importantly, 3503 (59%) protein spots were detected in two or more subjects with Sz, 3322 (58%) in two or more subjects with BD and 3211 (65%) in two or more of the control subjects (Figure 2).

**Figure 3:**
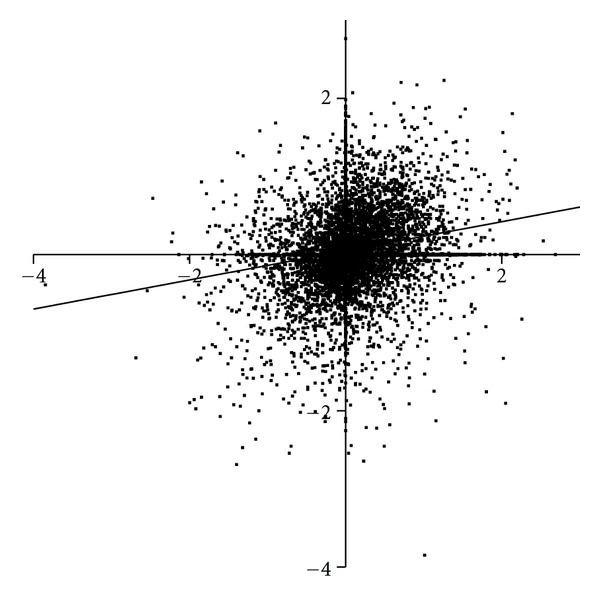
A comparison of the intensity of protein spots, normalised to the intensity of the corresponding protein spot in control CNS, in Brodmann's area 46 from subjects with Sz (Y-axis) and bipolar disorder (X-axis). Spots in the upper left quadrant show increased levels in Sz compared to bipolar disorder whereas those in the lower right quadrant show increased levels of proteins in bipolar disorder with lower levels in Sz.

The data from a simple modification of an established methodology serve to show how existing technologies can still have value in the study of the human CNS proteome. The increased utility of such modifications can be illustrated by comparing the intensity of each protein spot, for protein present in at least two cases, from subjects with Sz and BD normalised to the intensity of the same spot in the proteome of control subjects (Figure 3). These data show that differential changes in many relative protein intensities are occurring in the CNS of subjects of Sz and BD and the challenge is to mine this rich data set to identify candidate proteins that may prove valuable as biomarkers to separate the two disorders.

## 9. Conclusions

There are clear and urgent needs to develop biomarkers to aid in the diagnoses and monitoring of treatment in subjects with psychiatric disorders such as Sz [5]. It will be important not to grow impatient and prematurely discard the hypothesis that biomarkers can be identified to aid in the diagnoses and treatment of the disorder. For example, in diabetes, the development of clinically useful biomarkers has spanned several centuries from the discovery of sweet urine to immunological diagnostic tests for certain forms of diabetes [13]. Moreover, the recognition of the Federal Drug Authority that at least one pharmacogenomics test (based on genetic variation in cytochrome P450) is of value in assessing likely treatment responsiveness in people with Sz [102] suggest that progress is being made toward biomarker discovery, validation and eventually widespread clinical use. This being said, those in the biomarker research space need to be considering some form of standardisation of blood collection to attempt to minimise blood protein level variation due to factors other than disease pathophysiology or treatment response. In addition, those involved in biomarker research using postmortem CNS or neuroimaging need to develop networks that will facilitate a rapid validation of potential biomarkers across brain collections and between imaging centres. Both these strategies may be essential for the rigorous testing of potential biomarkers to occur rapidly as a prelude to moving such validated biomarkers into the clinic.

## Figures and Tables

**Table 1:** Proteins suggested as potential biomarkers for Sz or BPD due to diagnostic-specific changes detected using surface-enhanced laser desorption/ionization time of flight mass spectrometry in postmortem CNS.

Diagnoses	Proposed biomarker
Sz	CD58 molecule Calmodulin 1 (phosphorylase kinase, delta) Mago-nashi homolog, proliferation-associated (Drosophila) MYC associated factor X Regulator of G-protein signaling 11 Ubiquitin carboxyl-terminal hydroxylase isozyme L1 Cyclophilin A CEBPZ CCAAT/enhancer binding protein (C/EBP), zeta LOC153154 similar to short heat shock protein 60 Hsp60s2 Phosphoglycerate mutase 1 Tyrosine 3-monooxygenase/tryptophan 5-monooxygenase activation protein, eta polypeptide NK2 homeobox 4, 2,4-dienoyl CoA reductase 2 Peroxisomal, methylthioadenosine phosphorylase Purine nucleoside phosphorylase Bystin-like Ankyrin repeat domain 12 Proteasome (prosome, macropain) subunit, alpha type, 7 Aldolase C, fructose-bisphosphate Sphingomyelin phosphodiesterase 1, acid lysosomal Glutamate-ammonia ligase and enolase 2 (gamma, neuronal)

BPD	Myelin basic protein Dickkopf homolog 2 (Xenopus laevis) CEBPZ CCAAT/enhancer binding protein (C/EBP), zeta; 2,4-dienoyl CoA reductase 2, peroxisomal Bystin-like Ankyrin repeat domain 12 Aldolase C, fructose-bisphosphate
